# Clinical Benefit of Percutaneous Treatment of Fontan Pathway Obstructions

**DOI:** 10.3390/jcm15062240

**Published:** 2026-03-16

**Authors:** Anastasia Schleiger, Julia Moosmann, Damian Schaffner, Marie Schafstedde, Jan Brühning, Paul Spiesecke, Hans Peter Müller, Stanislav Ovroutski, Felix Berger, Peter Kramer

**Affiliations:** 1 Department of Congenital Heart Disease—Pediatric Cardiology, Deutsches Herzzentrum der Charité—Medical Heart Center of Charité, Augustenburger Platz 1, 13353 Berlin, Germany; julia.moosmann@dhzc-charite.de (J.M.); damian.schaffner@dhzc-charite.de (D.S.); marie.schafstedde@dhzc-charite.de (M.S.); jan.bruening@dhzc-charite.de (J.B.); stanislav.ovroutski@dhzc-charite.de (S.O.); felix.berger@dhzc-charite.de (F.B.); peter.kramer@dhzc-charite.de (P.K.); 2 Charité—Universitätsmedizin Berlin, Corporate Member of Freie Universität Berlin and Humboldt-Universität zu Berlin, 10117 Berlin, Germany; 3 Institute for Cardiovascular Computer-Assisted Medicine, Deutsches Herzzentrum der Charité—Medical Heart Center of Charité, Augustenburger Platz 1, 13353 Berlin, Germany; 4 Berlin Institute of Health, Charité—Universitätsmedizin Berlin, 10178 Berlin, Germany; 5 Department of Radiology, Charité—Universitätsmedizin Berlin, 10117 Berlin, Germany; paul.spiesecke@charite.de (P.S.); hans_peter.mueller@charite.de (H.P.M.); 6 German Center for Cardiovascular Research (DZHK), Partner Site Berlin, 10785 Berlin, Germany

**Keywords:** Fontan pathway obstruction, endovascular treatment, Fontan stenosis, Fontan stent implantation, physical capacity, Fontan-associated liver disease

## Abstract

**Background/Objectives:** Fontan pathway obstructions are a common complication during long-term follow-up after Fontan surgery. The clinical or hemodynamic benefit of percutaneous treatment of Fontan stenoses is poorly understood. In this study we analyzed the feasibility and clinical effects of percutaneous treatment of Fontan pathway obstructions. **Methods:** From April 2019 to December 2024 35 Fontan patients received percutaneous treatment of Fontan pathway obstructions by stent implantation. Indications for treatment included clinical signs of lower venous congestion or PLE and/or a moderate-to-severe morphologic pathway stenosis independent of clinical sequelae. Median follow-up time after the intervention was 1.5 years [IQR 0.7; 2.2]. **Results:** Median patient age was 20.3 years [IQR 16.3; 26.8]. Interventional success was defined as a significant increase in median indexed Fontan conduit cross sectional area and was achieved in all patients by expanding from 98.4 mm^2^/m^2^ [IQR 80.7; 115.5] to 145.1 mm^2^/m^2^ [IQR 134.8; 167.0, *p* < 0.001]. In symptomatic patients, a significant clinical improvement was detected 6 months after the intervention including an amelioration of physical capacity assessed by NYHA classification (*p* = 0.039) and cardiopulmonary exercise testing (VO_2peak_; *p* = 0.025). Global assessment of Fontan-associated liver disease (FALD) showed improvement during follow-up (*p* = 0.035). No peri- or postprocedural complications occurred. **Conclusions:** Percutaneous treatment of Fontan pathway obstruction has a high interventional success rate with a significant increase in indexed cross-sectional area. During follow-up, improvement of patients’ clinical condition and global signs of FALD were observed. The impact of percutaneous Fontan pathway obstruction relief on reversing or delaying the progression of FALD requires further investigation.

## 1. Introduction

The Fontan operation is the final-stage surgical palliation for patients with univentricular anatomy providing acceptable long-term survival [1,2]. The intracardiac lateral tunnel has a theoretical growth potential due to the included native atrium which is one of the rationales for performing this Fontan modification in some centers [3]. However, the lateral tunnel modification is associated with various morbidities which may also include the development of Fontan pathway obstructions caused by inadequate growth of the native atrium, scarring of the anastomoses or the baffle patch or compression by intravascular devices [4]. In most high volume pediatric cardiac centers, the extracardiac total cavopulmonary connection using an extracardiac prosthetic conduit is the preferred surgical technique [5,6]. Its introduction significantly simplified the surgical procedure, improved flow dynamics and reduced the onset of arrhythmias [7,8]. However, the frequently used polytetrafluoroethylene (Gore-Tex) grafts fail to accommodate somatic growth and additional neo-intima proliferation or thrombogenic potential of prosthetic material may result in Fontan conduit stenosis. Data describing the exact incidence and extent of Fontan pathway obstruction are rare, nonetheless a mean reduction in Fontan conduit cross-sectional area by 14–18% has been observed starting as early as 6 months after Fontan surgery [9,10]. A significant size mismatch between the Fontan conduit and the inferior vena cava (IVC) is associated with unfavorable flow dynamics leading to a 2–3-fold increase in kinetic energy due to acceleration and a significant energy loss at the IVC–conduit junction [11]. Additionally, energy loss caused by an unfortunate Fontan pathway geometry with reduced indexed Fontan diameters is aggravated during physical activity resulting in exercise intolerance [12]. Hence, eliminating vessel narrowing in the Fontan circulation might reduce energy loss in the conduit junction and increase physical capacity.

Despite improved survival rates during the past decades, Fontan-palliated patients are susceptible to various long-term complications [13]. Fontan-associated liver disease (FALD) represents one of the most prevalent forms of end-organ dysfunction, contributing substantially to increased morbidity and mortality [14,15]. The exact pathomechanisms of FALD development are not completely understood; however, hepatic venous congestion and diminished cardiac output have a considerable impact on its manifestation and progression [16]. Therefore, the relief of Fontan pathway obstructions and the elimination of IVC–conduit size-mismatch-induced hepatic congestion might delay the progression of hepatic deterioration.

This study aimed to evaluate the technical feasibility and interventional success of percutaneous stent implantation for Fontan pathway obstruction. Additionally, the clinical impact on physical capacity and the progression of FALD was assessed.

## 2. Materials and Methods

### 2.1. Study Design and Endpoints

All Fontan patients that received a percutaneous treatment of a Fontan pathway obstruction by stent implantation during the study period from April 2019 to December 2024 were included. The study was approved by the institutional review board and ethics committee (decision number: EA2/009/21). Informed consent was not mandatory due to the retrospective character of this study. Patients who developed a clinically significant Fontan stenosis early postoperative were excluded from analysis (*n* = 3). Patients aged <18 years were classified as pediatric and those ≥18 years as adults. Primary endpoints were procedural success and re-intervention rate. The procedure was considered successful with an increase in minimal indexed Fontan pathway cross-sectional area (CSA) ≥25% at the level of stenosis and the absence of a pressure gradient and major complications such as stent embolization or wall injuries. Minimal Fontan pathway CSA was calculated based on diameters in biplane angiography and indexed to patients` body surface area. Re-interventions were defined as unplanned secondary interventional procedures performed to treat re-stenosis or complications occurring after primary treatment. Secondary end-points were physical capacity, laboratory testing, hepatic ultrasound and shear wave elastography (SWE) and dispersion (SWD) values before and after percutaneous treatment. Physical capacity was assessed by New York Heart Association (NYHA) classification and cardiopulmonary exercise testing (CPET) using a cycle ergometer. SWE/-D measurements were performed during routine hepatic ultrasound by an experienced hepatologist or pediatric radiologist. The FALD score to assess overall FALD severity was determined as previously described [17]. Briefly, the FALD score was calculated based on results from three diagnostic modalities: laboratory testing, hepatic ultrasound and transient elastography. For score calculation, scoring points were assigned for each hepatic abnormality detected leading to a maximum of 15 points. Based on the median and percentiles of the FALD score the extent of FALD was categorized as mild (≤3 scoring points), moderate (4–5 scoring points) and severe (≥6 scoring points) [17]. Protein-losing enteropathy (PLE) was defined as a combination of persistent diarrhea and/or recurring edema and/or pleural effusions and/or ascites, decreased serum albumin (<3.5 g/dL) and total serum protein levels (<6.0 g/dL), and confirmation of intestinal protein loss with increased fecal alpha-1antitrypsin levels [18]. One patient with severe and therapy-resistant cholelithiasis was excluded from analysis of post-procedural hepatic follow-up.

### 2.2. Percutaneous Treatment of Fontan Pathway Obstruction

**Figure 1:**
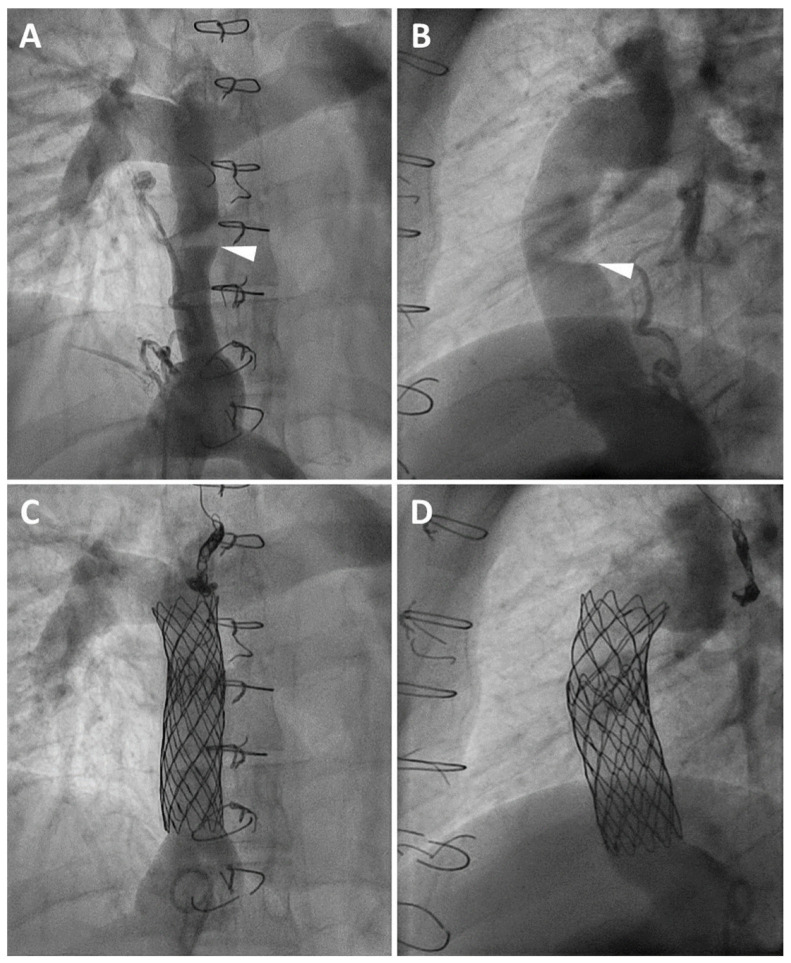
A.p. (**A**) and lateral (**B**) angiograms revealing a moderate-to-severe stenosis in the mid-portion of an 18 mm extracardiac conduit (white arrow) accompanied by a significant narrowing of the entire Fontan pathway in a 19-year old patient. Minimal Fontan conduit diameter ap: 5.8 mm, lateral: 8.2 mm (arrowheads). Vena cava inferior diameter a.p.: 32.4 mm, lateral: 22.4 mm. Minimal indexed cross-sectional area: 37.4 mm^2^/m^2^. (**C**,**D**): Treatment of Fontan conduit stenosis by implantation of two bare Cheatham-Platinum^TM^ stents (10-zig/50 mm and 8-zig/35 mm, postdilated with a 18 and 20 mm Atlas^TM^ Gold balloon). Minimal stent diameter a.p.: 17.6 mm, lateral: 18.6 mm. Minimal indexed cross-sectional area: 256.6 mm^2^/m^2^.

Indications for treatment included clinical signs of lower venous congestion such as edema, ascites, hepatosplenomegaly and/or PLE and/or impairment of physical capacity (*n* = 31) and/or a moderate-to-severe morphologic pathway stenosis independent of clinical sequelae (*n* = 4). A Fontan pathway stenosis was defined as moderate-to-severe when a significant luminal reduction of ≥25% of the internal diameter was present at the narrowest point of the extracardiac conduit or lateral tunnel on anterior–posterior and lateral angiographic projections, compared with the remaining angiographically measured conduit diameter (Figure 1A,B) [19,20]. Additionally, a pressure gradient ≥1 mmHg across the stenosis was considered significant and, in the presence of the above clinical or morphological criteria, would serve as an indication for treatment. In cases of lateral tunnel dilatation or in the presence of long-segment extracardiac tunnel narrowing, a lumen reduction exceeding ≥25% was defined in reference to the caliber of the nearest non-dilated segments of the Fontan pathway, rather than the over-dilated region itself. After obtaining hemodynamic measurements and performing biplane angiograms, a long delivery sheath was introduced into the Fontan pathway (12–16 F, Check-Flo Performer^®^ Introducer, Cook Medical Europe Ltd., Limerick, Ireland) using a 0.035 inch Amplatz Extra Stiff guidewire (Cook Medical Europe Ltd., Limerick, Ireland). Various stents have been implanted (Figure 1C,D) including Cheatham-Platinum^TM^ (NuMED, Hopkinton, MA, USA), AndraStent^®^ (Andramed GmbH, Reutlingen, Germany), Intrastent^TM^ Mega^TM^ LD/Max^TM^ LD (Medtronic, Minneapolis, MN, USA) or BeGraft aortic stents (Bentley InnoMed GmbH, Hechingen, Germany). Non-premounted stents were mounted on balloon-in-balloon catheters (BiB^®^, NuMED, Hopkinton, MA, USA) or occasionally on a Powerflex^TM^ balloon (Cordis, Nyon, Switzerland). Balloon diameter was selected by oversizing the original Fontan extracardiac conduit by 1–2 mm. In patients with a lateral tunnel dilatation, balloon diameters were determined based on the caliber of the nearest non-dilated segments of the Fontan pathway, excluding the over-dilated region. Stent length was chosen according to the morphology of the Fontan conduit stenosis. In patients with a patent fenestration the stent landing zone was selected either above or below it to avoid interference. In 6/35 patients (17.1%), two stents were implanted to achieve complete expansion along the length of the entire conduit. In the majority of the cohort, a post-dilatation was performed using 16–24 mm Atlas^TM^ Gold (Beckton, Dickinson and Company, Franklin Lakes, NJ, USA) or 26 mm Z-med II^TM^ balloons (NuMED, Hopkinton, NY, USA) to achieve complete stent expansion and ensure complete apposition to the inferior vena cava. All patients received continuous antithrombotic therapy with vitamin K antagonist or non-vitamin K antagonist oral anticoagulant (NOAC) after stent implantation.

### 2.3. Post-Procedural Follow-Up

Before discharge, all patients underwent a detailed clinical examination, echocardiography and chest x-ray. Short-term follow-up was performed 6 to 12 months after the intervention and included echocardiography, CPET, laboratory analysis and hepatic ultrasound including SWE/-D measurement.

### 2.4. Statistical Analysis

Data were obtained from institutional electronic medical records. Patients’ characteristics are expressed as median and interquartile range (IQR, 25th; 75th percentile) or frequency and percentage. Differences between groups were analyzed using the Chi-square test for categorical variables as well as the Wilcoxon rank sum test and Mann–Whitney U test for continuous variables. Statistical analysis was performed using GraphPad Prism statistical software program (GraphPad Software, Vers. 10.6.1, Boston, MA, USA). A *p*-value < 0.05 was considered statistically significant.

## 3. Results

### 3.1. Patients’ Characteristics

A total of 250 Fontan-palliated patients received a routine or clinically indicated cardiac catheterization at our institution during the study period. In 35/250 patients (14.0%) a percutaneous treatment of a Fontan pathway obstruction was performed by stent implantation. A comparative table of demographical and morphological features between patients with and without percutaneous stent implantation is provided in Table 1. Briefly, the majority of baseline characteristics significantly differed between Fontan stent recipients and non-recipients. Fontan stent recipients were characterized as follows: the median patient age at percutaneous treatment was 20.3 years [IQR 16.3; 26.8] and median time after the Fontan surgery was 16.4 years [IQR 12.4; 23.2]. The most common underlying morphologies were double-inlet left ventricle, tricuspid atresia or unbalanced atrioventricular septal defect. Fontan modifications included an extracardiac conduit in 31/35 patients (88.6%) and a lateral tunnel in 4/35 patients (11.4%). The distribution of Fontan modifications did not differ between treated and untreated cohorts (*p* = 0.606). The size of the implanted Fontan conduit varied from 16 to 24 mm, however conduits with a diameter of 18 mm were predominantly utilized. In the stent recipients, a patent fenestration was present in the minority of patients. Six patients subsequently underwent secondary device closure of the fenestration which had been performed previously using Amplatzer^®^ Septal Occluder (*n* = 5) or Amplatzer^®^ Muscular VSD Occluder (*n* = 1, Abbott, Chicago, IL, USA). Hemodynamic characteristics of Fontan stent recipients are provided in Table 2. Briefly, systolic ventricular function of the systemic ventricle was normal or mildly reduced in 26/35 patients (74.3%), whereas in 9/35 patients (25.7%) a moderate or severe impairment was observed. Atrioventricular valve regurgitation was classified as absent or mild in the majority of the cohort. Invasive evaluation revealed acceptable Fontan hemodynamics with regard to transpulmonary pressure gradient, pulmonary vascular resistance and end-diastolic pressure of the systemic ventricle. However, median pulmonary artery pressures and hepatic vein pressures were slightly elevated. PLE was present in 6/35 patients (17.1%) prior to stent implantation; 4/35 patients (11.4%) exhibited non-PLE-related peripheral edema or ascites.

### 3.2. Procedural Results

**Figure 2:**
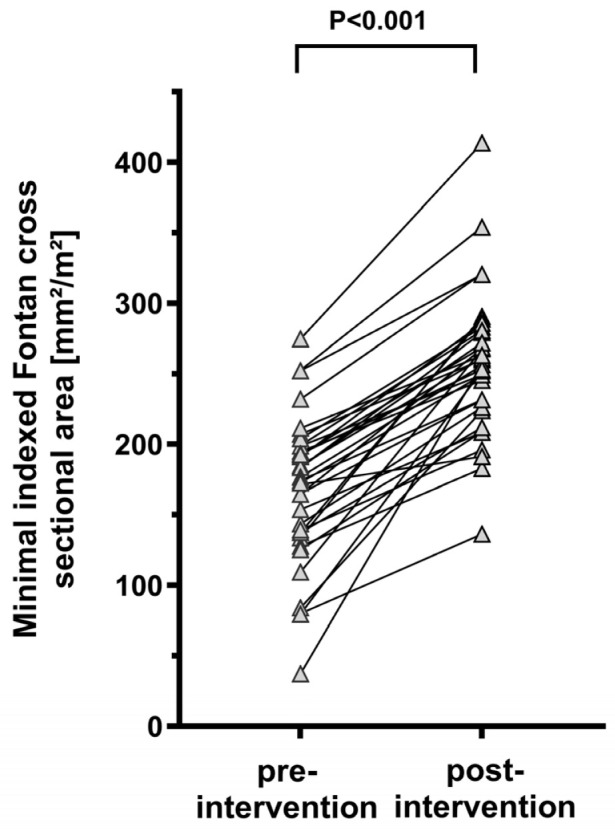
Point diagram depicting minimal indexed Fontan conduit cross-sectional area before and after percutaneous treatment (*n* = 35).

Thirty-one patients suffered from a slow but progressive decline in physical capacity as well as PLE, peripheral edema, ascites, weight gain or sonographic signs of moderate FALD. In four patients, Fontan pathway stenosis was diagnosed during routine follow-up examinations including echocardiography, cross-sectional imaging or invasive evaluation. In all treated patients, angiographies revealed at least a moderate Fontan pathway stenosis with a significant overall reduction in the expected median Fontan pathway CSA by 30.1% [26.2; 38.6]. Additionally, significant enlargement of the inferior vena cava and hepatic veins was observed (Table 3). The median invasive pressure gradient across the Fontan pathway obstruction was 1.0 mmHg [IQR 1.0; 2.0]. Fontan stenosis was detected at the caudal anastomosis in 21/35 patients (60.0%), at the cranial anastomosis in 5/35 patients (14.2%) and at the mid-portion of the Fontan pathway in 3/35 patients (8.6%). In 6/35 patients (17.1%) a complete narrowing of the Fontan pathway along its entire length was observed. A previously performed secondary device closure of the fenestration did not impact the development of Fontan pathway stenosis. Procedural success was achieved in all patients with a significant increase in median indexed Fontan pathway CSA from 98.4 mm^2^/m^2^ [IQR 80.7; 115.5] to 145.1 mm^2^/m^2^ [IQR 134.8; 167.0, *p* < 0.001], respectively (Figure 2). Additionally, a significant decline in the pressure gradient was detected (Table 3). No peri- or postprocedural complications such as stent embolization, dissection or stent restenosis occurred. Subgroup analyses stratified by ventricular dysfunction, the severity of atrioventricular valve regurgitation and Fontan pathway stenosis revealed no significant differences in post-interventional outcomes (all *p* > 0.05; Supplemental Table S1).

### 3.3. Post-Procedural Follow-Up

**Figure 3:**
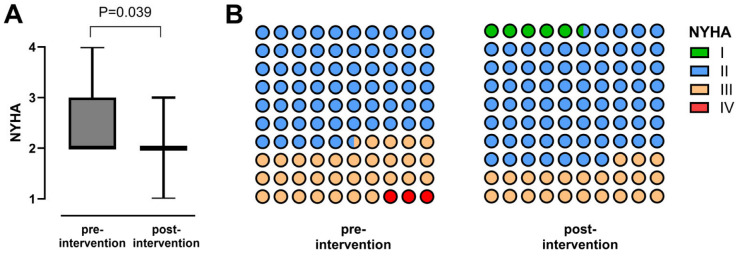
Box plot (**A**) depicting NYHA functional class before and after percutaneous treatment of Fontan conduit obstruction (*n* = 35). Colored point diagram (**B**) depicts frequency distribution of NYHA functional class before and after percutaneous treatment of Fontan stenosis. Green = NYHA I, blue = NYHA II, orange = NYHA III, red = NYHA IV. NYHA = New York Heart Association.

**Figure 4:**
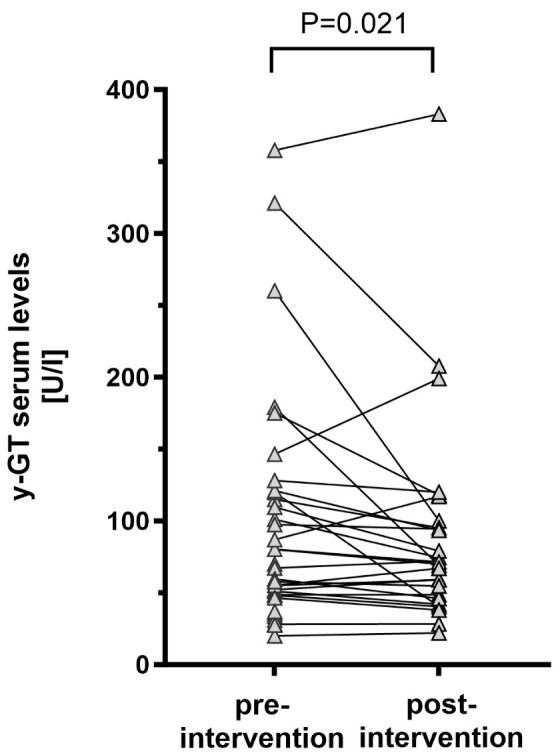
Point diagram depicts serum levels of γ-glutamyltransferase before and after percutaneous treatment of Fontan conduit obstruction (*n* = 34).

**Figure 5:**
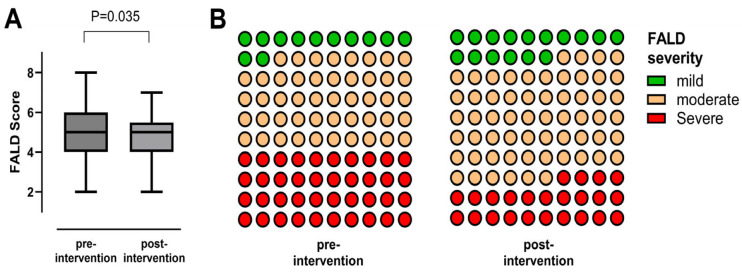
Box plot (**A**) depicting the FALD score before and after percutaneous treatment of Fontan conduit obstruction (*n* = 26). Colored point diagram (**B**) depicts frequency distribution of FALD severity before and after percutaneous treatment of Fontan stenosis. Green = mild, orange = moderate, red = severe. FALD = Fontan-associated liver disease.

Follow-up data were available in 34/35 patients (97.1%) with a median post-procedural follow-up time of 1.5 years [IQR 0.7; 2.2]. In symptomatic patients who presented with reduced physical capacity, NYHA functional class significantly improved during follow-up (Figure 3). Additionally, peak oxygen uptake (VO_2peak_) significantly increased (*p* = 0.025) and VE/VCO_2_ slope significantly decreased (*p* = 0.041) after Fontan stent implantation, while no statistically significant changes in O_2_ pulse were observed (Table 3). Serum levels of N-terminal prohormone of brain natriuretic peptide (NT-pro-BNP) or red cell distribution width (RDW) did not significantly change during follow-up. A significant increase in median transcutaneous oxygen saturation from 92.0% [IQR 91.0; 95.0] to 94.0% [IQR 92.0; 96.0] was detected (*p* = 0.007). In all patients who presented with symptomatic PLE or non-PLE-related peripheral edema or ascites, a significant clinical improvement was detected culminating in remission or relief of symptoms. Findings indicating hepatic congestion such as elevated γGT levels significantly decreased after percutaneous relief of Fontan pathway obstruction (Figure 4), whereas no significant changes in hepatic stiffness or dispersion values were detected. As assessed by the FALD score, global signs of FALD decreased slightly but statistically significant during follow-up (Figure 5).

In 10/35 patients (28.6%) a re-catheterization was performed after a median follow-up time of 1.5 years [IQR 1.5; 2.2]. Indications for re-catheterization comprised an elective re-evaluation of Fontan hemodynamics after stent implantation in 7/10 patients (70.0%) and a continuously reduced cardiopulmonary capacity in 3/10 patients (30.0%). A re-intervention was performed in 6/10 patients (60.0%): two patients received a planned postdilatation of the Fontan stent to achieve additional stent expansion and complete apposition of the proximal stent end to the inferior vena cava 6 to 12 months after initial stent implantation. Three patients received an unplanned balloon dilatation or stent implantation in the left pulmonary artery to treat a moderate-to-severe stenosis or restenosis and one patient received an unplanned embolization of a veno-venous collateral.

## 4. Discussion

In this study we retrospectively analyzed the interventional success and clinical results of percutaneous treatment of Fontan pathway obstructions with regard to symptoms of venous congestion, physical capacity and the progression of FALD. Our data indicate that percutaneous stent implantation is safe, efficient and significantly increases Fontan pathway diameters and indexed CSA.

The ideal size of the Fontan conduit at time of implantation remains controversial [21,22]. Due to the lack of growth potential, the implantation of conduits, which are compatible with the size of an adult IVC, might be desirable. However, excessively oversized conduits may lead to unfavorable Fontan hemodynamics based on a significant energy loss at the IVC–conduit junction and increased risk for thrombosis [7]. Independently from the initial conduit size implanted during Fontan surgery, a significant decrease in CSA is reported during mid-term follow-up [10]. The limited growth potential of the prosthetic material and its predisposition for circumferential neointimal proliferation and thrombus formation are major contributors for the development of obstruction and inadequately small diameters [10]. Considering that on average 70% of systemic venous return enters the pulmonary circulation via the IVC in adult Fontan patients, an adequate size and unrestricted blood flow are indispensable to optimize cardiac preload and output. Potential beneficial effects of a virtual expansion of 16 mm conduits to 24–32 mm diameters on Fontan hemodynamics and flow rates in adult patients have been shown in computational fluid dynamics modeling, suggesting that conduit sizes initially implanted in pediatric patients do not accommodate the increasing anatomic and hemodynamic requirements of an adolescent or adult [23]. Since various studies describe the enormous effects of Fontan conduit geometry on flow dynamics, energetic efficiency and energy dissipation, percutaneous relief of Fontan stenosis and size mismatch has come into focus during recent years [24,25]. Although various studies describe the feasibility and interventional success of the procedure, the clinical short- and long-term benefits of Fontan stent implantation remain unclear. Only a few studies with a limited number of patients report a significant improvement of NYHA functional class after percutaneous treatment of Fontan pathway stenosis while no changes in BNP levels, CPET parameters or non-invasive markers of FALD were observed [26,27]. Additionally, the optimal timing of this intervention to achieve maximum outcome with regard to delaying the onset of hemodynamic deterioration and the development of second organ disease has not yet been identified.

In our cohort, percutaneous relief of Fontan pathway stenosis diminished clinical signs of lower venous congestion such as ascites, edema and enteric protein loss independent from the extent of hemodynamic compromise. Additionally, in analogy with results from other investigators, the increase in indexed Fontan pathway CSA resulted in a significant improvement in physical capacity assessed by NYHA functional class [26]. This subjective increase in exercise tolerance was accompanied by a slight increase in the CPET parameter VO_2peak_. Additionally, the observed decrease in the parameter VE/VCO_2_ suggests an improvement in ventilatory efficiency which might be indirectly caused by an improved pulmonary perfusion.

Serum NT-pro-BNP levels and RDW, both biomarkers associated with heart failure severity, did not significantly change after percutaneous treatment. However, in a population characterized by progressive clinical and hemodynamic decline, the absence of worsening after intervention may already represent a clinically meaningful result. In addition, a significant increase in the transcutaneous oxygen saturation was detected after resolution of Fontan pathway stenosis, which may reflect improved pulmonary blood flow and enhanced Fontan pathway flow dynamics. Since cyanosis was identified as an independent risk factor for adverse outcome, a sustained elevation of oxygen saturation might be indicative of a benefit for the long-term outcome in these Fontan-palliated patients [28].

These findings underline the importance of determining the optimal timing for percutaneous relief of Fontan conduit obstructions to delay the onset of clinical deterioration. An adequate comprehensive patient surveillance including routinely performed cross-sectional imaging and invasive evaluation seems indispensable to detect abnormalities of the Fontan pathway geometry and initiate timely treatment.

### Fontan-Associated Liver Disease

FALD is the most frequent end-organ dysfunction following the Fontan operation and significantly increases morbidity and mortality [14,29]. The etiopathogenesis of FALD is not completely understood, however multiple factors seem to contribute to its development and progression [14,29]. In particular, compromised Fontan hemodynamics affect vascular supply and drainage of the liver and are related to the onset of hepatic damage [30]. With hepatic venous congestion and diminished cardiac output being considered as the major pathomechanisms of FALD, it seems intuitive that percutaneous treatment of Fontan pathway stenosis might ameliorate its development and progression. In patients with hepatic venous congestion, increased sinusoidal pressures cause decreased vascular supply to the intrahepatic bile ducts attributed to epithelial cell injury and release of γ-GT [30,31]. A significant reduction in γ-GT serum concentrations 6 to 12 months after the relief of the Fontan pathway obstruction might be indirectly considered as a successful mitigation of hepatic congestion. However, other indirect non-invasive diagnostic markers of congestive hepatopathy such as SWE/-D values or some sonographic signs of FALD did not significantly improve during follow-up. However, SWE and especially SWD, are not yet established diagnostic instruments for hepatic congestion in Fontan-palliated patients; its sensitivity and specificity for detection of venous congestion and hepatic fibrosis still have to be validated. Additionally, the majority of patients included in this study were characterized by an incipient deterioration of Fontan hemodynamics and a moderately advanced FALD. Impaired hemodynamics and pre-existing end-organ damage might explain the absence of a significant improvement in various parameters associated with FALD. Given the progressive nature of FALD, the absence of worsening on ultrasound findings itself might be considered a significant finding. Nevertheless, the FALD score, incorporating various parameters related to FALD, decreased slightly but was statistically significant in our cohort after percutaneous treatment of Fontan pathway obstructions, indicating improvement of FALD to some extent.

Since the reduction in Fontan pathway diameter and CSA have been reported to occur as early as six months after the Fontan surgery [9,10], an early invasive or non-invasive screening for Fontan pathway stenosis might be beneficial to provide the onset of hemodynamic and hepatic decline. Considering the safety and effectiveness of Fontan pathway stenting, an aggressive approach performing this intervention in early adolescence could be considered a preventive strategy. Various clinical and bench testing studies proved the possibility of a significant conduit expansion beyond nominal diameters to eliminate even the smallest increases in Fontan pathway resistance [32,33]. However, evidence-based guidelines or a consensus recommendation defining the indications for endovascular treatment of Fontan pathway stenosis in asymptomatic patients without a measurable pressure gradient are missing and treatment indications will remain a matter of debate in the absence of convincing longitudinal data. Since Fontan hemodynamics are altered under catheterization conditions using analgosedation, exercise hemodynamics might provide more reliable information concerning the hemodynamic relevance of a morphologic stenosis [34,35]. Rapid volume expansion is another valuable diagnostic instrument to unmask the hemodynamic significance of a Fontan pathway stenosis and can be readily implemented in the clinical practice [36,37].

Moreover, pre- and postinterventional computational fluid dynamics modeling to estimate dissipation and energy loss before and after Fontan stent implantation could provide more detailed information concerning the hemodynamic characteristics of a stenosis and therefore the hemodynamical benefits of this intervention in the future.

The evident knowledge gaps emphasize that further studies evaluating the optimal timing and the exact indications for Fontan stent implantation are indispensable.

## 5. Conclusions

In this study we demonstrated that percutaneous treatment of Fontan pathway obstruction is safe, efficient and effective. Stent implantation in the stenotic Fontan pathway successfully abolished clinical signs of lower venous obstruction, improved physical capacity and reduced several markers of FALD. However, additional studies are required to evaluate the preferable time point and long-term benefits of these interventions, in particular considering its impact on preventing or delaying the onset of hemodynamic deterioration and end-organ damage.

## 6. Limitations

There are several limitations to this study. This is a retrospective study with a comparably small sample size. The limited number of patients and the heterogeneity of the study cohort might distort the results of statistical analysis. The statistical power of the subgroup analysis was limited by the small sample size. Moreover, due to its retrospective character, CPET and hepatic assessment were not available in all patients, which might bias statistical evaluation. Additionally, the short duration of follow-up is insufficient to provide confident information concerning the long-term benefits of Fontan stent implantation.

## Figures and Tables

**Table 1:** Demographical and morphological characteristics of Fontan stent recipients and non-recipients.

Baseline Parameters	Fontan Stent Recipients (n = 35)	Fontan Stent Non-Recipients (n = 215)	*p*-Value
Patient age at procedure (years)	20.3 [16.3; 26.8]	13.1 [7.0; 22.4]	**<0.001**
Age at Fontan surgery (years)	3.7 [3.3; 6.9]	3.7 [2.8;5.1]	0.289
Gender, male	22/35 (62.9%)	110/215 (51.2%)	**<0.001**
Patient weight (kg)	63.0 [50.5; 77.0]	40.5 [21.0; 61.7]	**<0.001**
Underlying morphology			
DILV	8/35 (22.8%)	23/215 (10.7%)	**0.004**
uAVSD	7/35 (20.0%)	22/215 (10.2%)	
TA	8/35 (22.9%)	56/215 (26.0%)	
DORV	3/35 (8.6%)	18/215 (8.4%)	
HLHS	4/35 (11.4%)	58/215 (27.0%)	
Other	5/35 (14.3%)	38/215 (17.7%)	
Fontan modification			
APC	0/35 (0.0%)	2/215 (0.9%)	0.606
Extracardiac conduit	31/35 (88.5%)	179/215 (83.8%)	
Lateral tunnel	4/35 (11.4%)	34/215 (15.8%)	
Fontan conduit size (mm)			
8 *		1/169 (0.6%)	0.417
12 *		1/169 (0.6%)	
14 *		3/169 (1.8%)	
16	5/31 (16.1%)	31/169 (31.3%)	
18	18/31 (58.0%)	85/169 (50.3%)	
20	6/31 (19.4%)	39/169 (23.1%)	
22	0/31 (0.0%)	8/169 (4.7%)	
24	2/31 (6.4%)	1/169 (0.6%)	
Patent fenestration	4/35 (11.4%)	89/215 (41.4%)	**0.004**
Minimal Fontan pathway diameter (a.p., mm)	12.8 [10.9; 14.0]	14.9 [13.5; 16.5]	**<0.001**
Minimal Fontan pathway diameter (lat., mm)	17.7 [17.0; 18.2]	18.1 [16.3; 20.1]	**0.047**
IVC diameter (a.p., mm)	26.2 [21.8; 30.1]	20.9 [16.6; 25.2]	**<0.001**
IVC diameter (lat., mm)	25.6 [22.6; 29.1]	19.3 [16.5; 23.7]	**<0.001**
Minimal indexed Fontan cross sectional area (mm^2^/m^2^)	98.4 [80.7; 115.5]	183.2 [133.8; 249.2]	**<0.001**
% deviation, predicted vs. measured indexed Fontan conduit cross sectional area	–30.1 [−26.2; −38.6]	−20.4 [−15.9; −26.9]	**<0.001**

Data are presented as median and interquartile range [IQR] or frequency and percentage. a.p. = anterior–posterior; DILV = Double-inlet left ventricle; DORV = Double-outlet right ventricle; HLHS = Hypoplastic left heart syndrome; lat. = lateral; TA = Tricuspid atresia; uAVSD = Unbalanced atrioventricular septal defect; IVC = Inferior vena cava. * Hepatic vein conduit. Values with *p* < 0.05 were considered statistically significant and are shown in bold.

**Table 2:** Hemodynamic and anatomic characteristics of Fontan stent recipients.

Anatomic Parameters	Median [IQR] or Frequency (%)
Nakata Index (mm^2^/m^2^)	236.0 [181.0; 284.5]
LLI (mm^2^/m^2^)	156.0 [132.5;213.5]
**Hemodynamic parameters**	
Impairment of systolic ventricular	
function	
None/mild	26/35 (74.3%)
Moderate	8/35 (22.9%)
Severe	1/35 (2.9%)
AV valve regurgitation	
None/mild	30/35 (85.7%)
Moderate	5/35 (14.2%)
**Invasive hemodynamic parameters**	
mPAP (mmHg)	12.0 [10.0; 15.0]
SVEDP (mmHg)	10.0 [6.0; 16]
TPG (mmHg)	4.0 [3.0; 4.0]
HVP (mmHg)	14.0 [10.5; 15.0]
HVWP (mmHg)	14.0 [11.3; 16.7]
CO (L/min)	4.2 [3.4; 4.6]
CI (L/min/m^2^)	2.5 [2.0; 2.8]
PVR (WU)	1.0 [0.7; 1.4]
PVRi (WU×m^2^)	1.8 [1.4; 2.3]

Data are presented as median and interquartile range [IQR] or frequency and percentage. CI = Cardiac index; CO = Cardiac output; HVP = Hepatic vein pressure; HVWP = Hepatic vein wedge pressure; LLI = Lower Lobe Index; mPAP = Mean pulmonary artery pressure; PVR = Pulmonary vascular resistance; PVRi = Pulmonary vascular resistance index; SVEDP = Systemic ventricle end-diastolic pressure; TPG = Transpulmonary pressure gradient.

**Table 3:** Morphological and clinical changes after percutaneous treatment of Fontan pathway obstruction.

Hemodynamic Parameters	Pre- Intervention	Post- Intervention	*p*-Value
Pressure gradient across Fontan stenosis (mmHg)	1.0 [1.0; 2.0]	0.0 [0.0;0.0]	**<0.001**
NT-proBNP (pg/mL)	132.6 [64.7; 253.2]	92.15 [47.8; 318.1]	0.760
RDW (%)	13.6 [12.6;15.7]	13.7 [12.9; 15.9]	0.446
Transcutaneous oxygen saturation (%)	92.0 [91.0; 95.0]	94.0 [92.0; 96.0]	**0.007**
CPET parameters *			
VO_2peak_ (ml/kg/min) *	21.0 [17.0; 23.2]	22.7 [13.6; 29.7]	**0.025**
O_2_ pulse (ml/beat) *	9.2 [8.1; 11.6]	10.9 [7.1; 12.6]	0.267
VE/VCO_2_ slope *	32.9 [30.5; 38.0]	30.9 [28.0; 33.5]	**0.041**
**Morphological parameters**			
Minimal diameter Fontan stenosis (mm, a.p.)	12.8 [10.9; 14.0]	17.1 [14.6; 19.1]	**<0.001**
Minimal diameter Fontan stenosis (mm, lat.)	17.7 [17.0; 18.2]	18.6 [17.3; 19.6]	**0.004**
Suprahepatic IVC diameter (mm, a.p.)	26.2 [21.8; 30.1]	27.7 [26.2; 29.7]	0.194
Suprahepatic IVC diameter (mm, lat.)	25.6 [22.6; 29.1]	26.1 [22.5; 29.0]	0.754
**Hepatic assessment**			
Thrombocyte count (K/ul) ^#^	166.5 [136.0; 221.3]	183.0 [137.0; 229.5]	0.139
Albumin (mg/dL)	4.5 [4.0; 4.9]	4.5 [4.0; 4.9]	0.721
GOT (U/L) ^#^	30.0 [22.8, 34.9]	30.4 [25.5; 37.0]	0.948
GPT (U/L) ^#^	29.0 [21.2; 37.5]	29.3 [21.9; 41.6]	0.503
Bilirubin (mg/dL) ^#^	0.8 [0.6; 1.2]	0.7 [0.5; 1.1]	0.164
Hepatic ultrasound findings ^†^			
Hepatomegaly ^#^	11/26 (42.3%)	10/26 (38.5%)	0.778
Splenomegaly ^#^	9/26 (34.6%)	9/26 (34.6%)	1.000
Abnormal parenchyma structure ^#^	25/26 (96.2%)	25/26 (96.2%)	1.000
Segmental atrophy/hypertrophy ^#^	3/26 (11.5%)	3/26 (11.5%)	1.000
Hepatic vein dilatation	18/26 (69.2%)	19/26 (73.1%)	0.705
Abnormal hepatic vein architecture ^#^	5/26 (19.2%)	5/26 (19.2%)	1.000
Hyperechogenic lesions ^#^	11/26 (42.3)	11/26 (42.3)	1.000
Ascites ^#^	4/26 (15.3%)	2/26 (7.7%)	0.385
Surface nodularity ^#^	3/26 (11.5%)	2/26 (7.7%)	0.760
TE (kPA) ^#;†^	12.1 [9.8; 17.8]	12.2 [10.2; 14.0]	0.131
SWE (kPa) ^‡^	12.0 [9.9; 19.5]	11.4 [9.8; 13.7]	0.131
SWD ((m/s)/kHz) ^‡^	17.6 [15.9; 21.6]	17.8 [15.7; 19.0]	0.119

Data are presented as median and interquartile range [IQR] or frequency and percentage. a.p. = Anterior–posterior; CPET = Cardiopulmonary exercise testing; GOT = Glutamate oxaloacetate transaminase; GPT = Glutamate-pyruvate transaminase; IVC = Inferior vena cava; lat. = Lateral; NT-proBNP = N-terminal pro b-type natriuretic peptide; RDW = Red cell distribution width; SWD = Shear wave dispersion; SWE = Shear wave elastography; TE = Transient elastography; VE = Minute ventilation, VCO_2_ = Carbon dioxide output; VO_2_peak = Peak oxygen uptake. * Only data from patients with both pre- and postprocedural CPET analysis (*n* = 17/35) were included for analysis ^†^ Only data from patients with both pre- and postprocedural ultrasound examinations (*n* = 26/35) were included for analysis ^‡^ Only data from patients with both pre- and postprocedural SWE (*n* = 17/35) and SWD (*n* = 16/35) values were included for analysis ^#^ FALD score parameters. Values with *P* < 0.05 were considered statistically significant and are shown in bold.

## Data Availability

The data that support the findings of this study are available on request from the corresponding author, (A.S.). The data are not publicly available due to privacy and ethical restrictions.

## Supplementary Materials

The following supporting information can be downloaded at: https://www.mdpi.com/article/10.3390/jcm15062240/s1. Table S1: Pre-to Postinterventional changes stratified by ventricular dysfunction, atrioventricular valve insufficiency and severity of Fontan pathway stenosis.

## Abbreviations

The following abbreviations are used in this manuscript:

CSA	Cross-sectional area
CPET	Cardiopulmonary exercise testing
FALD	Fontan-associated liver disease
γGT	γ-Glutamyltransferase
IQR	Interquartile range
IVC	Inferior vena cava
mPAP	Mean pulmonary artery pressure
NOAC	Non-vitamin K antagonist oral anticoagulant
NT-pro-BNP	N-terminal prohormone of brain natriuretic peptide
NYHA	New York Heart association
PLE	Protein-losing enteropathy
RDW	Red cell distribution width
SWD	Shear wave dispersion
SWE	Shear wave elastography
VO_2peak_	Peak oxygen uptake
