# Supplementary material for: Clinical Benefit of Percutaneous Treatment of Fontan Pathway Obstructions
Source: J Clin Med. 2026 Mar 16;15(6):2240. doi: 10.3390/jcm15062240 (PMC13026709; doi:10.3390/jcm15062240)
Supplement: Supplementary file 1 [file jcm-15-02240-s001.zip › jcm-4200106-supplementary.pdf]

Supplemental Table 1. Pre-to Postinterventional changes stratified by ventricular dysfunction, atrioventricular valve insufficiency and severity of Fontan pathway stenosis

Parameter	No/Mild VD (n=27)	Moderate/severe VD (n=8)	*P*-Value	No/mild AVVI (n=30)	Moderate/severe AVVI (n=5)	*P*-Value	Mild FPS (n=8)	Moderate/severe FPS (n=27)	*P*-Value
ΔNT-proBNP (pg/ml)	0.0 [-56.0; 46.2]	-14.1 [-69.9; 53.0]	0.770	2.1 [-44.0; 51.9]	-108.6 [-252.5; 199.9]	0.142	-3.0 [-173.5; 61.3]	0.0 [-50.2; 43.3]	0.823
ΔRDW (%)	-0.3 [-0.8; 0.4]	0.0 [-0.8; 1.3]	0.470	-0.2 [-0.6; 0.4]	-0.3 [-3.8; 0.3]	0.680	-0.1 [-1.8; 1.1]	-0.2 [-0.8; 0.4]	0.928
ΔThrombocyte count (K/ul)	-16.0 [-23.5; 13.0]	-6.0 [-16.0; 5.0]	0.760	-3.0 [-18.5; 9.8]	-21.0 [-35.5; -9.0]	0.106	-17.0 [-31.0; -6.0]	-1.0 [19.3; 13.0]	0.176
ΔAlbumin (mg/dl)	0.1 [-0.3; 0.5]	0.0 [-0.4; 0.3]	0.682	0.2 [-0.3; 0.5]	-0.2 [-0.7; 0.1]	0.125	0.1 [-0.7; 1.1]	0.1 [-0.3; 0.5]	0.925
ΔGOT (U/l)	0.0 [-6.1; 5.0]	1.5 [-0.1; 18.4]	0.319	0.5 [-6.0; 5.1]	0.8 [-4.2; 14.5]	0.729	-5.9 [-6.5; -0.1]	1.3 [-2.2; 5.3]	0.092
ΔGPT (U/l)	0.5 [-6.4; 7.7]	2.0 [-1.9; 10.0]	0.346	1.2 [-5.7; 7.3]	2.0 [-7.2; 28.3]	0.483	-1.9 [-7.0; 5.6]	1.8 [-5.6; 8.2]	0.386
ΔγGT (U/l)	8.0 [-3.9; 16.6]	32.4 [-4.1; 87.1]	0.360	8.2 [-0.3; 24.8]	-3.9 [-30.1; 94.7]	0.603	-7.4 [-52.7; 7.4]	8.7 [-0.7; 28.3]	0.073
ΔBilirubin (mg/dl)	0.1 [-0.4; 0.3]	-0.1 [-0.2; 0.2]	0.265	0.7 [-0.2; 0.3]	0.1 [-0.1; 0.4]	0.696	-0.0 [-0.1; 0.5]	0.1 [-0.2; 0.2]	0.894
ΔTCS (%)	-2.0 [-4.0; 0.0]	1.0 [-4.0; 3.0]	0.268	-2.0 [-4.0; 0.0]	-3.0 [3.0; 4.0]	0.569	-4.0 [-4.0; -2.0]	-1.0 [-3.0; 0.0]	0.085
ΔVO_{2peak} (ml/kg/min)	-3.7 [-0.8; 0.4]	-1.5 [4.2; 1.7]	0.448	-2.1 [-6.8; 1.2]	-2.6 [3.2; 0.3]	0.874	-2.9 [-6.2; 2.5]	-2.4 [-6.3; 0.7]	0.808
ΔO_2 pulse (ml/beat)	-0.3 [-2.3; 0.1]	0.7 [-3.1; 3.3]	0.643	-0.3 [-1.8; 0.2]	-0.1 [-3.5; -0.1]	1.0	-1.7 [-2.8; -1.7]	-0.4 [-2.2; 0.2]	1.0
ΔVE/VCO_2 slope	3.8 [-2.4; 6.9]	1.5 [-1.8; 6.8]	0.173	3.0 [-1.8; 0.2]	4.9 [1.5; 4.9]	0.497	6.0 [0.5; 6.0]	2.6 [-3.3; 6.2]	0.312
ΔFALD Score	0.0 [0.0; 1.0]	1.0 [0.0; 2.0]	0.167	0.0 [0.0; 1.0]	1.0 [1.0; 3.0]	0.437	0.0 [0.0; 1.0]	1.0 [0.0; 1.0]	0.718

Data are presented as median and interquartile range [IQR]. Δ was calculated as preintervention minus postintervention values.

AVVI = atrioventricular valve insufficiency; FALD = Fontan-associated liver disease; FPS = Fontan pathway stenosis; γGT = Gamma-glutamyl transferase; GOT = glutamate oxaloacetate transaminase; GPT = glutamate-pyruvate transaminase; NT-proBNP = N-terminal pro b-type natriuretic peptide; RDW = Red blood distribution width; TCS = transcutaneous saturation; VD = ventricular dysfunction; VE = minute ventilation, VCO_2 = Carbon dioxide output; VO_2peak = peak oxygen uptake.

*
